# Pylephlebitis and Hepatic Abscess Secondary to Complicated Appendicitis in a Geriatric Patient With Undiagnosed Situs Inversus Totalis: A Case Report

**DOI:** 10.7759/cureus.97664

**Published:** 2025-11-24

**Authors:** Christian Ballardo Medina, Mariana Burgueño Ibarra, Paul Humberto Valdez Castillejo, Jose Manuel Rocha Chavez, Rodolfo Lopez Hernandez, Ana Karen Carrasco Gaxiola

**Affiliations:** 1 General Surgery, Instituto de Seguridad y Servicios Sociales de los Trabajadores del Estado (ISSSTE) Universidad Autónoma de Sinaloa (UAS), Culiacán, MEX

**Keywords:** complicated appendicitis, exploratory laparotomy, geriatric, intra-abdominal abscess, situs inversus

## Abstract

Pylephlebitis is an uncommon but severe complication of intra-abdominal infections, particularly associated with conditions such as appendicitis or diverticulitis. Although it is more frequently observed in older men, it can occur in other age groups and carries significant mortality if not diagnosed in a timely manner. Acute appendicitis remains the leading cause of emergency abdominal surgery worldwide, with a higher risk of complications in elderly patients due to diagnostic challenges. Situs inversus totalis, an unusual anatomical condition, may delay diagnosis by altering the clinical location of symptoms. We report the case of a geriatric patient with previously undiagnosed situs inversus totalis who developed complicated appendicitis with pylephlebitis and hepatic abscess, highlighting the clinical and therapeutic challenges of this combination.

## Introduction

Pylephlebitis, or septic thrombosis of the portal vein, is a rare but serious condition secondary to abdominal infections that drain into the portal system. Its incidence ranges between 0.37 and 2.7 cases per 100,000 inhabitants, with a predominance in men over 50 years of age. The main associated pathologies are diverticulitis (26%), appendicitis (22%), and inflammatory bowel disease (6%). In up to 8% of cases, a hepatic abscess may coexist, although it is not always clear whether it acts as a cause or a consequence. Prompt diagnosis is essential because mortality rises to 14% compared to 8% in uncomplicated appendicitis [1,2].

Acute appendicitis is the leading cause of non-traumatic urgent abdominal surgery worldwide, with the highest incidence among young adults. In patients over 65 years of age, atypical clinical presentation and age-related physiological changes increase complication and mortality rates, which can reach up to 8% compared to 1% in younger patients. In elderly individuals, there is a higher rate of preoperative diagnostic error compared to younger populations [3]. Complicated appendicitis in elderly patients ranges from 18% to 70%, with perforation (35%) and abscess being the most common complications. It is frequently associated with treatment delays due to diagnostic difficulties caused by vascular, immunological, and histological changes, which alter the clinical picture and reduce the accuracy of commonly used scoring systems [2]. Anatomical variations, such as situs inversus totalis (approximate incidence of 1 in 100,000), alter the usual localization of thoracoabdominal organs, increasing the risk of diagnostic errors and the probability of complications such as pylephlebitis or hepatic abscesses [1-5].

The aim of this study is to review the literature on complicated appendicitis in patients with situs inversus totalis and to present a clinical case managed at the Regional Hospital 'Dr. Manuel Cárdenas de la Vega', Culiacán, Mexico.

## Case presentation

A 78-year-old male presented with no history of surgical or chronic degenerative diseases. He reported a history of tobacco, alcohol, and marijuana use. The patient described the onset of his condition 20 days prior to admission to a secondary care hospital, with left hypochondrial pain radiating to the epigastrium, described as dull and continuous, associated with a 5-kg weight loss, abdominal distension, and melena. He was started on proton pump inhibitors and antibiotics, with partial improvement and subsequent discharge. Eight days later, he was readmitted to the same unit with persistent symptoms. Laboratory studies revealed thrombocytosis (700,000 platelets/microliters (µL); reference: 150,000-400,000), coagulation abnormalities (prothrombin time 23.7 seconds; reference: 11-13.5; international normalized index (INR) 2.41; reference: 0.8-1.2), and elevated alkaline phosphatase (310 units (U)/Liter (L); reference: 44-147 U/L) (Table 1).

**Table 1:** Laboratory study values.

Laboratory tests	Reference value	Patient values
Platelets	150,000-400,000 platelets/µL	700,000 platelets/µL
Prothrombin time	11-13.5 seconds	23.7 seconds
Alkaline phosphatase	44-147 U/L	310 U/L

**Figure 1:**
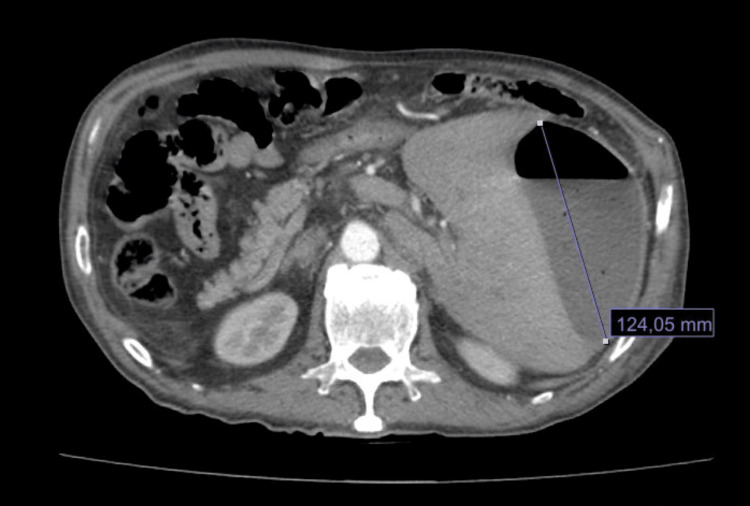
Axial CT scan with IV contrast showing hepatic abscess and hepatomegaly. The presence of situs inversus is observed.

**Figure 2:**
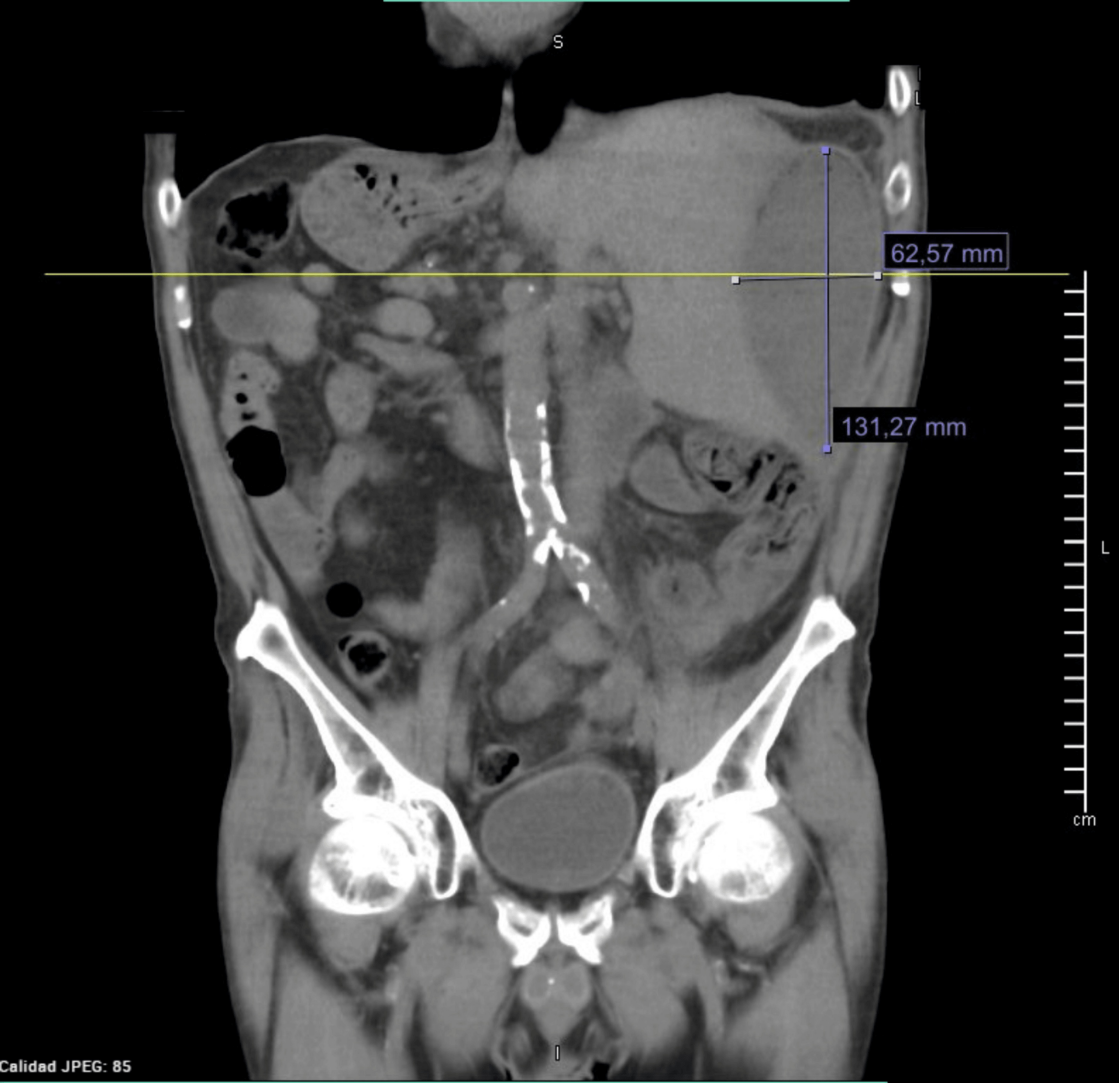
Coronal CT scan with IV contrast showing a hepatic abscess measuring 131.27 × 62.57 mm.

**Figure 3:**
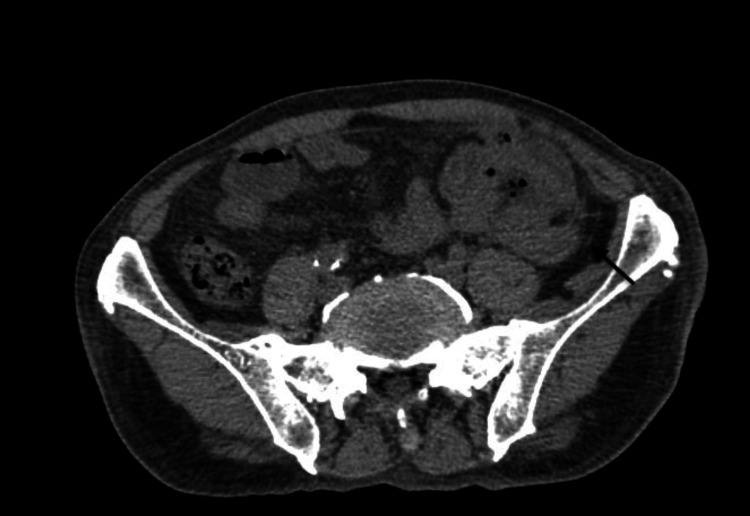
Axial CT scan showing a phlegmon in the left iliac fossa with associated pericolic fat stranding (black arrow).

Abdominal ultrasound and contrast-enhanced computed tomography (CT) reported situs inversus totalis, hepatomegaly, portal vein dilation (16 millimeters (mm)), mild ascites, and a perihepatic collection of 140 cubic centimeters (cc) (Figures 1-2). He was referred to our tertiary care hospital for management. One week later, percutaneous drainage of the perihepatic collection adjacent to the round ligament was performed, yielding 400 cc of purulent material. Empirical antibiotic therapy with ceftriaxone and metronidazole was initiated. Culture of the drained material was positive for *Escherichia coli​​​​​*. Despite treatment, the patient’s clinical course was poor, and a surgical consultation was requested. A follow-up CT scan revealed fat stranding in the left iliac fossa without evidence of hepatic collection (Figure 3). An exploratory laparotomy was performed, revealing a phlegmon in the left iliac fossa, a necrotic appendix perforated at the cecal base with a fecalith (stage IV appendicitis), and a 1-centimeter (cm) ileal perforation near the ileocecal valve. A left hemicolectomy (15 cm of ileum, cecum, ascending colon, and one-third of the transverse colon) with ileostomy was performed. The postoperative course was uneventful, and he was discharged after seven days of continued antibiotic therapy. One year later, an ileotransverse side-to-side isoperistaltic anastomosis was performed using a 60 mm blue Endo GIA stapler (Medtronic PLC, Minneapolis, Minnesota, USA)​​​​​​, with closure and reinforcement of the defect using Connell-Mayo and Lembert sutures (polydioxanone (PDS); Ethicon, Inc., Somerville, New Jersey, USA; size 3-0). The patient tolerated liquids on postoperative day 3 and was discharged on day 5 without complications.

## Discussion

Pylephlebitis is a rare but severe complication of intra-abdominal infections, significantly increasing morbidity and mortality in acute abdominal conditions. In this case, we report a 78-year-old male with stage IV complicated appendicitis associated with pylephlebitis, hepatic abscess, and situs inversus totalis - a very rare combination that complicated both diagnosis and treatment.

The reported incidence of pylephlebitis is low (0.37-2.7 cases per 100,000), primarily affecting men over 50, as in this case. Complicated appendicitis represented a clear risk factor, particularly due to delayed diagnosis and treatment. Situs inversus totalis further complicated the scenario by reversing the location of thoracoabdominal organs, making the typical presentation of right iliac fossa pain absent. Instead, the patient presented with left-sided abdominal pain, leading to an initial misdiagnosis of gastric pathology and symptomatic treatment rather than definitive management [1].

Progression to pylephlebitis with hepatic abscess emphasizes the importance of early evaluation with appropriate imaging in patients with risk factors and atypical symptoms. Hepatic abscesses are rare pathologies, with an incidence of approximately 0.0023% in the general population, and can be classified as pyogenic, amebic, or fungal. The characteristic clinical presentation includes fever, right upper quadrant pain, nausea, and weight loss. According to studies, pyogenic hepatic abscess secondary to acute appendicitis occurs in only 1% of cases, with CT being the best diagnostic tool. Standard management is drainage, usually guided by CT, ranging from single aspiration for abscesses smaller than 5 cm to catheter drainage for larger collections [5-7]. In this case, CT revealed indirect signs of portal hypertension and a perihepatic collection consistent with an abscess, appropriately managed with percutaneous drainage. However, diagnostic and surgical delays - 36 days from the first medical consultation - allowed progression to complicated appendicitis with phlegmon, necrosis, ileal perforation, and the need for extended right hemicolectomy with ileostomy. In elderly patients, appendicitis complication rates can reach 70% due to age-related immunological, vascular, and anatomical changes that alter clinical presentation and reduce the sensitivity of diagnostic tools [4].

Additionally, the patient’s history of smoking, chronic alcohol consumption, prolonged drug use, and limited access to healthcare likely contributed to immunological impairment, atypical clinical presentation, and delayed medical care, worsening the disease course.

Ultimately, surgical management was successful, and one-year follow-up confirmed good evolution without recurrence, with restoration of intestinal continuity. This case highlights the importance of early diagnosis, clinical suspicion in high-risk populations, and the need for appropriate diagnostic and surgical resources, particularly in regions with limited healthcare access.

## Conclusions

The geriatric patient represents a diagnostic challenge, particularly due to atypical clinical presentation, age-related physiological changes, and coexisting conditions such as situs inversus totalis, as demonstrated in this case. This case emphasizes the importance of maintaining a high index of suspicion for atypical abdominal symptoms, especially in patients with comorbidities and limited healthcare access. Delayed diagnosis and treatment of acute surgical conditions can lead to severe complications and major surgery, with morbidity and mortality rates reaching up to 18%.

Timely multidisciplinary management, proper use of imaging studies, and early surgical intervention are key to improving outcomes in these patients. Likewise, it is essential to promote equitable access to healthcare and strengthen clinical training regarding unusual pathologies to prevent avoidable adverse outcomes.
